# Follow-up between 6 and 24 months after discharge from treatment for severe acute malnutrition in children aged 6-59 months: A systematic review

**DOI:** 10.1371/journal.pone.0202053

**Published:** 2018-08-30

**Authors:** Natasha Phillipa O’Sullivan, Natasha Lelijveld, Alexandra Rutishauser-Perera, Marko Kerac, Philip James

**Affiliations:** 1 Department of Population Health, London School of Hygiene and Tropical Medicine, London, United Kingdom; 2 Brighton and Sussex Medical School, Falmer, East Sussex, United Kingdom; 3 Action Against Hunger, London, United Kingdom; 4 Centre for Maternal, Adolescent, Reproductive, and Child Health (MARCH), London School of Hygiene and Tropical Medicine, London, United Kingdom; 5 Medical Research Council (MRC) Unit The Gambia at the London School of Hygiene & Tropical Medicine, London, United Kingdom; TNO, NETHERLANDS

## Abstract

**Background:**

Severe acute malnutrition (SAM) is a major global health problem affecting some 16.9 million children under five. Little is known about what happens to children 6–24 months post-discharge as this window often falls through the gap between studies on SFPs and those focusing on longer-term effects.

**Methods:**

A protocol was registered on PROSPERO (PROSPERO 2017:CRD42017065650). Embase, Global Health and MEDLINE In-Process and Non-Indexed Citations were systematically searched with terms related to SAM, nutritional intervention and follow-up between June and August 2017. Studies were selected if they included children who experienced an episode of SAM, received a therapeutic feeding intervention, were discharged as cured and presented any outcome from follow-up between 6–24 months later.

**Results:**

3,691 articles were retrieved from the search, 55 full-texts were screened and seven met the inclusion criteria. Loss-to-follow-up, mortality, relapse, morbidity and anthropometry were outcomes reported. Between 0.0% and 45.1% of cohorts were lost-to-follow-up. Of those discharged as nutritionally cured, mortality ranged from 0.06% to 10.4% at an average of 12 months post-discharge. Relapse was inconsistently defined, measured, and reported, ranging from 0% to 6.3%. Two studies reported improved weight-for-height z-scores, whilst three studies that reported height-for-age z-scores found either limited or no improvement.

**Conclusions:**

Overall, there is a scarcity of studies that follow-up children 6–24 months post-discharge from SAM treatment. Limited data that exists suggest that children may exhibit sustained vulnerability even after achieving nutritional cure, including heightened mortality and morbidity risk and persistent stunting. Prospective cohort studies assessing a wider range of outcomes in children post-SAM treatment are a priority, as are intervention studies exploring how to improve post-SAM outcomes and identify high-risk children.

## Introduction

Severe acute malnutrition (SAM) is a major public health problem, estimated to affect 16.9 million children under 5 across the globe in 2017, causing over 516,000 child deaths per year [1, 2]. Estimating numbers of cases of SAM are obtained from prevalence surveys with an incidence correction factor applied. Calculating incidence using longitudinal data would provide a more accurate estimate of the caseload of SAM and mortality attributable to it. However, this type of data is rarely collected and therefore these figures likely underestimate the real burden of SAM [3]. The World Health Organisation’s (WHO) case definition of SAM specifies the presence of at least one of these three independent criteria: <-3 z-scores weight-for-height (WHZ), mid-upper arm circumference (MUAC) of <115mm, and presence of bilateral pitting oedema (also known as ‘kwashiorkor’) [4].

Current WHO management protocols are public-health focused and include: an emphasis on high programme coverage; early identification of affected children, allowing those with an appetite and who are clinically stable (i.e. ‘uncomplicated’ SAM) to be managed as outpatients using Ready-to-Use Therapeutic Food (RUTF); and inpatient care for the much smaller number of children with additional medical complications [5, 6]. This ‘Community-Management of Acute Malnutrition’ (CMAM) model of care is cost-effective, allows for increased caseloads, and is now supported by substantial evidence of having contributed to lower mortality rates from SAM [6–9].

Whilst there are currently no universal performance targets for CMAM programmes, the minimum standards set by the SPHERE humanitarian charter are globally recognised and widely used as basic targets for mortality, defaulter and recovery rates [10]. These are all short-term outcomes and contribute to many CMAM evaluations focusing on short-term successes only. Longer-term outcomes also matter and, if different to short-term outcomes, may indicate the need for additional care after the initial treatment episode. The wider perspective of CMAM outcomes is often neglected, despite evidence of high post-treatment mortality highlighted by a literature review in 2012 [11].

The WHO protocol for the management of SAM states that after discharge from treatment programmes children should be periodically monitored to avoid relapse, but has no clear definition of relapse nor does it provide specific guidance on how often and by whom this should be performed [6]. In some settings, successful rehabilitation from SAM is followed by referral for a further period of nutritional support in a supplementary feeding programme (SFP). Since SFPs are primarily for the treatment of moderate acute malnutrition (MAM) and are often run by different organisations to those running the CMAM programme, this can involve several challenges. SFPs are not universally available, data linkages are often weak so it may not be possible to know whether a child attended and how he/she fared on SFP, and SFP management protocols have a weaker evidence base than those for SAM [12–15].

A post-SAM follow-up period of 6–24 months is particularly neglected as the current evidence base tends to focus on short-term outcomes (<3 months after treatment for SAM) and longer-term outcomes (>24 months after treatment). Studies focusing on shorter-term outcomes document high levels of mortality and deterioration to MAM and low rates of sustained recovery [16–19]. Studies exploring longer-term outcomes in older children find evidence for increased stunting, reduced lean muscle mass, weaker hand grip and changes in electroencephalogram activity [20–22].

To date, there is no systematic review of outcomes at follow-up between 6–24 months after discharge from treatment for SAM in the literature. We aimed to identify and synthesise the current evidence on the outcomes of children who have been followed-up 6–24 months post-discharge from treatment for SAM using a systematic search strategy. Through highlighting our current knowledge and current evidence gaps in this area we hope to inform future programmes, research, and policy in order to improve sustained recovery and help children not only survive, but thrive after an episode of SAM.

## Methods

### Search strategy

Our protocol was registered on PROSPERO (PROSPERO 2017:CRD42017065650) on 11^th^ May 2017 (**S1 File**). We conducted a systematic literature search in Global Health, Embase and MEDLINE In-Process and Non-indexed citations databases between June and July 2017 with the last search on 19^th^ July. However, search alerts were turned on and new articles screened for eligibility until 28^th^ August, Search strategy was formulated using search terms and subject headings for 1) severe acute malnutrition, 2) child/infant, 3) therapeutic feeding intervention, 4) synonyms for the outcome parameter “follow-up”; using Medical Subject Headings and free text terms. No date limits were applied. Detailed information on the combinations of search terms used in our search strategy is shown in **S2 File**.

### Study selection

Inclusion criteria were studies in which children aged 6–59 months in a Low- or Middle-Income Country experienced an episode of SAM, received an inpatient or outpatient therapeutic feeding intervention for the treatment of SAM, were subsequently discharged as cured, and who presented any clearly defined outcome between 6 and 24 months of follow-up after discharge. Definitions of SAM included in this review were <-3 WHZ or <70% weight-for-height (WFH) using the 1977 National Centre of Health Statistics (NCHS) reference median or the 2006 WHO Growth Standards, MUAC< 115mm, MUAC <110mm, bipedal-oedema or clinical diagnosis of marasmus, kwashiorkor or marasmic-kwashiorkor [23, 24]. Discharge criteria for cured was as per study definition.

Exclusion criteria were unpublished studies or grey literature, reviews, non-human studies, studies focusing on management of SAM in infants <6 months, studies in high-income countries and studies not published in English language or published in abstract form only.

### Screening process

Titles and abstracts were screened against a screening form by the lead author only. The full text was then evaluated for complete suitability and included in review if it qualified. Data extraction forms were piloted then used on all studies to collect information on study details and outcomes. The standardized data extraction form is available in **S3 File.** Risk of bias was assessed at the study level using ‘Methods for the development of NICE public health guidance’ [25, 26].

Due to the heterogeneous nature of study designs, participants and outcomes, it was not meaningful to synthesize the results in a meta-analysis. The PRISMA (Preferred Reporting Items for Systematic Reviews and Meta-Analyses) guideline was followed, except for the items relating to meta-analysis (**S1 Checklist**).

## Results

**Fig 1:**
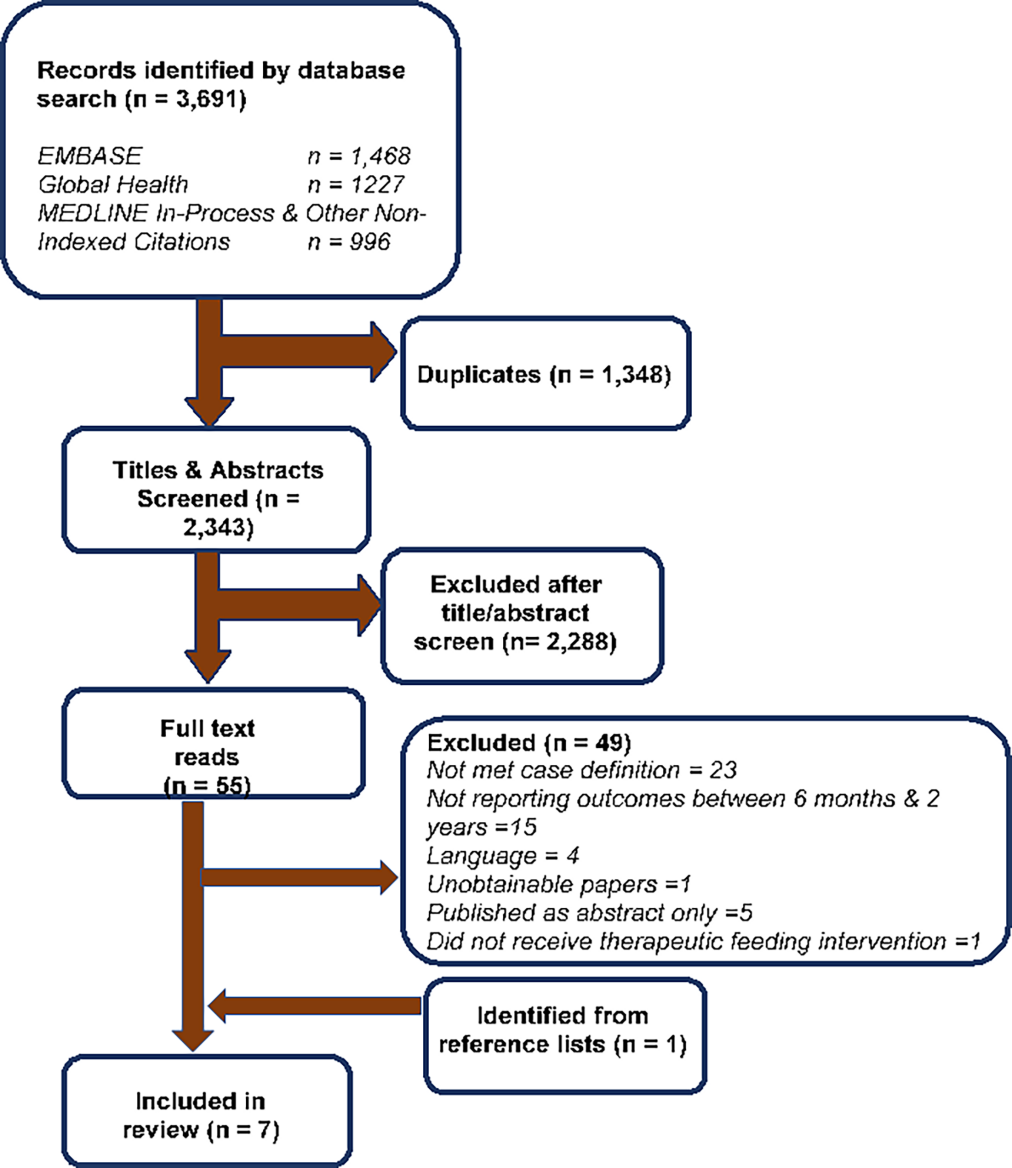
PRIMSA flow diagram.

We screened 3,691 records, ultimately identifying seven eligible articles following full-text review and searching of reference lists (**Fig 1**) [27–33]. Table 1 presents a summary of included studies’ results. The most common reason for exclusion (23 studies) was not meeting the case definition of SAM. A table of some of these excluded studies with the most similar admission criterion to our specification is available in **S1 Table.** Fifteen studies were excluded as outcomes reported did not fall into our pre-defined period of 6 to 24 months. Three of the studies took place in India, two in Malawi and one each in Burkina Faso and Bangladesh. All seven had different admission criteria and four of them had different discharge criteria. The quality assessment of the studies is available in **S2 Table.**

**Table 1:** Summary of results of studies included in review.

Title, Author, Year, Study Design	Population & Setting	Number discharged as cured	Admission (A) & Discharge (D) Criteria	Description of intervention	Description and timing (Months, mo) of Follow-Up	Outcomes reported & findings in children discharged as cured
Management of Kwashiorkor in its Milieu- A follow-up for Fifteen Months	All Under-5s (n = 1799) in 20 villages surveyed.	32	A: Clinical diagnosis of kwashiorkor	Outpatient	Monthly weight, height and diet recorded for 15mo.	Loss-to-follow-up: 0/32 (0%)
Khare, RD *et al*. [31]	Undertaken at the Rural Health Unit at a hospital in Mumbai, India		D: Oedema resolved and weight 65% of local growth chart reference levels	Local foods	Outcomes presented at 15mo	Mortality: 2/32 (6%)
1976						Relapse: 2/32 (6%)
Prospective cohort study						Morbidity: 25/32 (78%) helminthiasis 16/32 (50%) signs of Vitamin A Deficiency 14/32 (44%) infection of some kind 12/32 (38%) tichuriasis 2/32 (6%) tuberculosis 32/32 (100%) anaemic
Growth, morbidity, and mortality of children in Dhaka after treatment for severe malnutrition: a prospective study	12–59 months	437	A1: <60% weight-for-height (WFH) (NCHS)	Children had previously been enrolled in a randomised trial with three arms: inpatient, day-care or domiciliary-care	Visited at home every two weeks for 12mo.	**Loss-to-follow-up:** 102/437(23%) 33/102 (33%) lost with no trace 4/102 (4%) excluded due to tuberculosis 47/102 (46%) excluded as incomplete follow-up (<18 visits)
Khanum, S *et al*. [33]	All children admitted to nutrition rehabilitation unit (NRU) between December 1990 and November 1991 Dhaka, Bangladesh		A2: Bipedal oedema	Milk-based feeds and salt-free meals	Outcomes presented at 12mo.	**Mortality**: 10/437 (2%) 8/10 (80%) female
1998			D: ≥80% WFH median (NCHS growth references)			**Relapse:** 3/437 (0.6%)
Controlled trial						**Readmissions due to medical emergency**: 5/437 (1%)
						**Morbidity**: Illness recorded in 35% fieldworkers’ fortnightly visits. 308/335 (92%) experienced diarrhoea over the 12mo. Mean episodes: 7 (range: 0–30). 322/335 (96%) experienced cough with fever
						**Anthropometry** (change from discharge to end of follow-up at 12 mo.): Weight-for-height z-score (WHZ)→ from -1.6 to -0.9 Weight-for-age z-score (WAZ)→ from -3.7 to -3.1 Height-for-age z-score (HAZ)→no change overall, remained at -4.1
Uptake of HIV testing and outcomes within a community-based therapeutic care (CTC) programme to treat severe malnutrition in Malawi: a descriptive study	Average age at admission = 30 months (SD 17.2)	1783	A1: Bipedal-oedema	Inpatient/Outpatient CMAM	Children previously discharged from CMAM invited to participate.	**Loss-to-follow-up**: 441/1783 (25%) 180/1783 (10%) moved 113/1783 (6%) wrong address 148/1783 (8%) did not attend
Bahwere, P *et al*. [30]	All under-5s who had received CMAM living in the catchment area of 17 health centres in Dowa, Malawi		A2: MUAC<110mm	Weekly take-home Ready-to-use-therapeutic-food (RUTF)	Outcomes presented at median 15.6mo after discharge.	**Mortality**: 69/1783 (3.87%) 8/69 (12%) malaria 7/69 (10%) HIV 7/69 (10%) malnutrition 3/69 (4%) pneumonia
2008			A3: Marasmus			**Relapse** = 26/1783 (2%)
Retrospective cohort study			A4: Other criteria e.g. twins			
			D: >80% WFH (NCHS) +no bilateral-oedema			
Follow-up of post-discharge growth and mortality after treatment for severe acute malnutrition (FuSAM Study): a prospective cohort study	6–59 months	471	A1: WFH<70% NCHS	All initial stabilization as inpatient (~3 weeks), then transferred to outpatient therapeutic programme (OTP) (~10 weeks)	Ward-based review on 1-year anniversary of discharge date from OTP or followed-up at home >12mo post-discharge.	**Loss-to-follow-up:** 57/471 (12%)
Kerac, M *et al*. [27]	1024 children admitted to NRU in Blantyre, Malawi between July 2006 and March 2007.		A2: MUAC<110mm	F75 during stabilisation, RUTF in OTP.	Outcomes presented at 1-year follow-up.	**Mortality:** 49/471 (10%)
2014			A3: Bipedal oedema		Matched with one or more sibling controls identified at follow-up	**Relapse**: 7/471 (2%)
Cohort with matched controls			D: >80% WFH (NCHS), no oedema, clinically stable on 2 consecutive visits			**Anthropometry** (change from discharge to 12 months): WHZ→1.92 (95% CI 1.76,2.08) improvement to 0.04 (SD 1.0). Comparable to sibling controls WAZ→ 1.66 (1.50,1.82) improvement to -1.77 (1.1). Mean difference vs. siblings: -0.55 (-0.71, -0.3; p<0.01) HAZ→ 0.37 (0.21, 0.53) improvement to -2.97 (1.3) (p<0.01). Mean difference vs. siblings: -1.13 (-1.34, -0.93; p<0.01)
Socioepidemiological determinants of severe acute malnutrition and effectiveness of nutritional rehabilitation centre in its management	6–59 months	91	A1: WHZ <-3 (WHO Growth Standards) A2: Bipedal-oedema A3: MUAC <115mm	Inpatient	Called for follow-up 1, 2, 3 and 6mo after-discharge	**Loss-to-follow-up**: 27/91 (30%)
Aprameya, HS *et al*. [28]	Admitted to NRU between May 2013 and May 2014 in Mangalore, India.		D1: 5g/kg/day weight gain for 3 consecutive days D2: Active &alert D3: Absence bilateral-oedema, fever or infection D4: Tolerating home-based feeds D5: Caretaker confident to take child home	F75 for initial 2 days then F-100 with rice/lentils/porridge+ 1 egg/day	Outcomes presented at 6mo	**Mortality**: 4/91 (4%) 1/4 (25%) ventricular septal defect 1/4 (25%) bronchopneumonia 2/4 (50%) cerebral palsy
2015	Admissions referred by rural health workers or identified at outpatient department			Discharged at day 14		**Recovered** (WHZ≥-1) = 38/91 (42%)
Prospective cohort study	Group 1 = SAM purely due to dietary deficiency Group 2 = SAM secondary to chronic infections or underlying systemic infections					**Not recovered** (WHZ <-1 and ≥-3) = 22/91 (24%) 7/22 (32%) Group 1 15/22 (68%) Group 2 Difference: p = 0.023
						**Relapse** = 0/91 (0.0%)
						**Morbidity** = 39/91 (43%) experienced recurrent respiratory illness 14/91 (15%) experienced recurrent diarrhoeal illness
						**Weight gain** (Mean weight (kg) at discharge and 6mo) = Group 1 9.4 (2.0 SD) → 10.3 (2.2) Group 2 8.1 (2.4) → 8.9 (2.6) Difference: p<0.001
Seasonal effect and long-term nutritional status following exit from a community-based management of severe acute malnutrition program in Bihar, India	6–59 months	1659	A1: MUAC <115mm	Inpatient/Outpatient CMAM	Children discharged 3, 6, 9, 12 or 18mo previously were traced (different cohorts)	**Loss-to-follow-up**: 395/1659 (24%)
Burza,S *et al*. [29]	2667 children received CMAM between February 2009 and September 2011 in Bihar, India		A2: Bipedal oedema	RUTF	Outcomes presented for these periods combined	**Mortality**: 10/1659 (1%)
2016			D: MUAC >120mm, no oedema for 1 week+ clinically well and good appetite for 2 consecutive visits			**Relapse into SAM**: 45/1659 (2.7%)
Retrospective cohort study						**Number with moderate acute malnutrition:** 302/1659 (18%)
						**Anthropometry** (from discharge to 12m follow-up): HAZ→ improved by 0.7 (0.5,0.9, p<0.001)
Relapses from acute malnutrition and related factors in a community-based management programme in Burkina Faso	6–59 months	82	A1: MUAC<110mm	Outpatient CMAM (uncomplicated SAM only)	Children discharged from CMAM 6-20mo previously identified	**Loss-to-follow-up**: 37/82 (45%)
Somasse,YE *e al*. [32]	Children received CMAM for SAM and MAM between July 2010 and June 2011 in Burkina Faso		A2: Bipedal oedema	Weekly take-home RUTF		**Mortality**: 7/82 (9%)
2016	Identified via one-stage stratified and clustered sampling		D: WHZ >-2 z-scores (WHO Growth Standards) & no oedema			
Retrospective cohort study						

### Loss-to-follow-up

Loss-to-follow-up ranged from 0.0% to 45.1%. Aprameya *et al*. identified that distance from nutritional rehabilitation unit (NRU), travelling and food expenses, and loss of daily wages reduced compliance with follow-up visits [28]. Two studies found differences in baseline characteristics of children lost-to-follow-up. Losses and intermittent follow-up were more common in children who had been inpatients (p = 0.003) in Khanum *et al*. [33], and children lost-to-follow-up were more likely to be admitted with a smaller MUAC in Somasse *et al*. [32].

### Mortality

Mortality was calculated solely for the period of 6–24 months after discharge from therapeutic feeding programmes, despite many studies reporting a combined total of in-programme and after-discharge mortality. The highest number of post-discharge deaths reported was 10.4% (49/471) in Kerac *et al* [27]. The lowest mortality reported was 0.6% (10/1659) by Burza *et al*. [29]; but when we consider only those who were traced successfully, this increases to 7.9% (10/1264), similar to the 8.5% (7/82) seen in Somasse *et al*. [32].

Post-discharge mortality of treatment programmes in Asia was generally lower compared to those operating in Africa. CMAM programmes reported lower mortality compared to inpatient programmes operating in the same continent. However, the number of studies is too small to decipher if this is significant.

### Relapse

There were multiple discrepancies between the studies’ definition of relapse, and the timing of its measurement. Some studies reported relapse as a point prevalence, based on a single data collection point, whilst others reported incidence based on multiple data collection points. In Khanum *et al*., 0.6% (3/437) re-met the admission criteria at 1-year post-discharge. In Khare *et al*. 6.3% (2/32) had re-experienced SAM during the 15 months, but none of the children were experiencing it at the time of follow-up. In Aprameya *et al*. children were defined as recovered (defined as WHZ ≥1), or not recovered (WHZ ≥-3 but <-1) at 6 months. 24.1% (22/91) were classified as not recovered, and there were more children in this group who had SAM due to an underlying systemic illness than from poor nutrition (p = 0.023) [28]. 41.7% (38/91) of the children were defined as having recovered. At a single measurement 15 months after discharge in Bahwere *et al*. 26/1783 (1.5%) were malnourished (WFH <80% reference median and/or bilateral pitting oedema). Although not included in their definition of relapse, one additional child had a MUAC <110mm, and five had MUAC <125mm. There were stark differences in relapse rates according to HIV serostatus; 14.3% (4/28) in HIV+ compared to 2.0% (22/1094) in HIV-, in those children with known outcomes. 2.7% (45/1659) of all children discharged as cured met the criteria for SAM admission (MUAC<115mm and/or bipedal oedema) at a single measurement either 3, 6, 9, 12, or 18 months (average 9 months) post-discharge in Burza *et al*. [29]. As was done with mortality, when it is calculated for only children with known outcomes this increases to 3.6% (45/1264). When also including the case definition for MAM, 27.5% (347/1264) relapsed to SAM or MAM at an average follow-up time of 9 months.

### Morbidity

In Aprameya *et al*. 42.9% (39/91) and 15% (14/91) of children post-discharge had recurrent respiratory or diarrhoea illnesses during follow-up (28). 43.8% (14/32) in Khare *et al*. had some form of infection, 100% (32/32) were anaemic and 78.1% (25/32) had helminthiasis [31]. 402/437 (92%) in Khanum *et al*. experienced at least one episode of diarrhoea over the course of the 12-month follow-up, but the mean was seven episodes (range = 0–30) [33]. 292/437 (67%) attended an outpatient health centre with diarrhoea (105/437 (24%) at least three times), 319/437 (73%) with fever (114/437 (26%) at least three times) and 254/437 (58%) with pneumonia (44/437 (10%) at least three times). 420/437 (96%) reported a cough with fever; although this was less frequent in the domiciliary care group (p = 0.03). However, the domiciliary care group had an older age at admission (29 months vs. 25 and 26 months for inpatient and day-care groups, respectively) which could be a factor in this.

### Anthropometry

Kerac *et al*. found that from discharge to 12-months children underwent large catch-up in WHZ [27]. A mean improvement in WHZ of 1.92 (95% CI: 1.76, 2.08) brought them to a mean value of 0.04 WHZ at 12 months post-discharge; comparable to sibling controls and the global norm. WAZ also improved by 1.66 (1.50, 1.82) giving a mean value of -1.77. However, this was below that of sibling controls by -0.55 WAZ (-0.71, -0.38; p<0.01). This could be explained by HAZ, as there was only a small mean improvement of 0.37 (0.21, 0.53, p<0.01) to reach a final value of -2.97 (1.3 SD), below that of the sibling controls by -1.13 (-1.34, -0.93; p<0.01). Burza *et al*. noted similarly small levels of improvement in HAZ: a 0.7 (0.5, 0.9, p<0.001) increase from discharge to 12-months [29].

Khanum *et al*. also observed increases in mean WHZ and WAZ from discharge to 12-months of -1.6 to -0.9 and -3.70 to -3.08, respectively [33]. Mean HAZ did not improve over follow-up and remained at -4.14; classified as severe stunting. No associations were found between time with diarrhoea, fever or cough and levels of weight or height gain.

In Aprameya *et al*. there was an increase in mean weight from 9.2kg (2.0 SD) at discharge to 10.3kg (2.2) at 12-months for children without underlying illness (p<0.001) [28]. The same increase was observed for children with underlying illnesses from 8.1kg (2.4) to 8.9kg (2.6) (p<0.001), albeit lower at each time point. The difference in weight gain between groups was also significant (p<0.001).

## Discussion

Our systematic search of the literature on post-SAM treatment outcomes highlighted that there is very limited data reporting what happens to children after successful initial cure. In the existing studies there is marked diversity in the study design, population, HIV prevalence, admission and discharge criteria, nutritional intervention provided and definition of relapse, which make it challenging to directly compare studies. Despite the great variability between different studies and settings, our results suggest that despite nutritional recovery, some affected children remain vulnerable 6–24 months later, manifested through a variety of outcomes.

### Mortality

Routine SAM programme monitoring would have missed the post-discharge mortality that we observed. It is important to consider that the mortality reported here was calculated solely for the period of 6–24 months post-discharge (depending on the length of study follow-up) following nutritional cure, and does not include in-programme mortality. Mortality even among this group of “healthy survivors” is thus striking and is likely to reflect the fact that some children who present with SAM have pre-existing vulnerabilities, whereby SAM is manifested as a symptom and/or sign of those vulnerabilities, rather than due to poor dietary quality and/or quantity alone. Therapeutic feeding is thus sometime analogous to treating infection-related fever: an important part of the “package” of care but not necessarily addressing the root issue. Aside from infection (which is already treated in existing therapeutic feeding programmes) many other possible causes of poor growth are recognised which can be difficult to identify and address even in resource-rich settings, for example biological causes such as malabsorption, metabolic disorders and disability, and social causes such as neglect (either deliberate or, more often, due to complex family circumstances such as illness and poverty) [34, 35].

The highest post-treatment mortality reported by Kerac *et al*. of 10.4% is above even the in-programme SPHERE standards of <10% mortality [27]. This reflects the initial high-risk cohort: children were all originally admitted to inpatient care in 2006–2007, when CMAM services were unavailable locally and hence cases tended towards the ‘complicated’ end of the SAM spectrum. Malawi also has a high background child mortality as well as a high prevalence of HIV (43.5% (445/1024) of the original cohort of admissions were HIV seropositive) [36]. Another study in Malawi which followed-up children for four months after discharge found that HIV seropositive children were nearly three times more likely to die during nutritional rehabilitation; a 35.4% mortality risk compared to 10.4% (p<0.001) [37].

### Relapse

The current lack of a standard definition for post-discharge relapse was evident in these studies. The inconsistency in the definition and measurement of relapse means it is largely not possible to compare differences in, and burden of relapse across the different contexts. Although relapse reported in studies included in this review range from 0% to 6%, many other studies with shorter follow-up periods cite much higher figures. A study in Ethiopia found that 14 weeks after admission into an OTP, 34.6% had relapsed to SAM and a further 37.5% had MAM [17]. Relapse to SAM was 11.1% measured at monthly follow-up visits over the course of 6 months in the Democratic Republic of Congo, and this increased to 44.2% when relapse to MAM is included [38].

### Morbidity

High levels of morbidity, in particular diarrhoeal and respiratory illnesses, were reported in all three studies that measured morbidity [28, 31, 33]. A study in Ethiopia that followed-up children six months post-discharge found that there was approximately a 1.7 times higher incidence of fever, diarrhoea and cough in children discharged as cured compared to controls [39]. The synergistic effect of malnutrition and infection on risk of mortality is well-documented, and this review furthermore highlights the potential need for additional care for recovered malnourished children in order to prevent infection, as vulnerability to infection is still present despite recovery in WHZ [40].

### Anthropometry

Both included studies that measured WHZ at 6–24 months showed that children were no longer wasted [27, 33]. This has been observed by multiple other studies, including a Bangladesh follow-up that found WHZ increased from -2.5 (0.6 SD) at discharge to -1.9 (1.0) at 6 months [21, 41, 42]. A study from India that followed-up children between one and three, and five and seven years after an episode of SAM found that their mean WHZ was higher than their siblings who had not experienced malnutrition [21]. Whilst it is reassuring that complete or near-complete catch-up in WHZ is possible, there are potentially important life-course implications for these children as suggested by the accumulation of evidence on the Developmental Origins of Health and Disease (DOHAD) highlighting associations between rapid weight gain in infancy and later risk of non-communicable diseases (NCDs) [43].

Overall good recovery of WHZ needs to be seen in context of less impressive changes in other anthropometric indicators. All three studies included in this review that measured HAZ found there were, at best, limited improvements, which is consistent with the wider literature [27, 29, 33, 42, 44–46]. The lack of catch-up in HAZ is a significant concern and of critical importance as severely stunted children are 5.5 times more likely to die, and furthermore are 12.3 times more likely to die when any degree of stunting and wasting occur concurrently [47, 48]. Long term adverse health associated with stunting has been well described and includes an increased risk of suboptimal cognitive and physical development and risk of adult NCDs [49]. A recent Technical Briefing Paper on the relationship between wasting and stunting has helped the international community begin to recognise the gap in both knowledge and programming between these two forms of malnutrition, but this remains a problem to be addressed [50].

### Limitations

The use of different admission criteria (WHO versus NCHS; WHZ versus MUAC), which do not overlap perfectly, identifying similar but not the same children, has likely resulted in the subsequent study populations having variable initial risks of mortality and morbidity [51–53]. This issue was compounded by the use of different discharge criteria which resulted in distinct groups of children classified as recovered, making it hard to differentiate between post-discharge mortality and morbidity in recovered children and those who never achieved nutritional rehabilitation (had they been in a different programme).

Relatively high losses to follow-up also limit what can be learnt from our review. Of children not found at 6–24 months post-treatment, some will have died, some will be alive but unwell or still malnourished, and some will be alive and well [54]. What percentage are in each category is likely to vary programme to programme, but what is almost certain is that known mortality and morbidity are underestimates of the true value.

There is a significant risk of bias across all the studies in the review. Firstly, survivor bias will have likely affected all the studies and is critical to their interpretation. Only the healthiest children of those originally admitted for SAM would have made it to nutritional cure in the first place. It is therefore particularly striking that some of these are still at risk of mortality and morbidity over six months post-nutritional cure.

Kerac *et al*. was the only study which employed a control group to assess relative risk of observed outcomes: siblings living in the same household [27]. However, although 90% of siblings had never experienced SAM, they may have exhibited a degree of undernourishment themselves, which could reduce the effect size. In addition, HIV status was not known or reported on in all studies; despite being an important risk factor for poor outcomes in African studies [55]. In the absence of control groups in all studies except for Kerac *et al*., we cannot estimate the relative risk of our outcomes and the attributable risk of these related to a previous SAM episode. Despite being unable to say whether there is an excess of e.g. mortality and stunting among children after an episode of SAM, we feel the observed high rates give some cause for concern.

We must also be aware of other variables that influence the extent to which the study results are generalisable. For example, the infectious disease burden, health facility coverage, and food security status of a local population are factors that may influence the outcomes considered in this review, and therefore necessitate context-specific interpretation. Such factors need to be measured and analysed in future studies to better assess the associations between being treated for SAM and the observed outcomes in these cohort studies.

The final constraint when it comes to critical appraisal of the studies in this review is the lack of targets or standard literature to assess whether and to what extent observed mortality and morbidity represents an excess over that in the general population. All settings in which SAM is a public health problem are by definition high-risk. Without more background information on reference population mortality and morbidity, it is hard to quantify the excess risks in our post-SAM children. More context-specific information would be helpful to assess the additional level of support required for SAM survivors.

There are several methodological limitations of the review which warrant mention. Restricting to English language, published papers in the search strategy means not all relevant research may have been identified, and omission of important studies remains a possibility. Selection or reviewer bias could also have occurred given studies were not screened or abstracted in duplicate.

### Programmatic recommendations

Our results highlight the urgent requirement of a uniform definition of relapse. Once defined a simple method to enable programs to assess relapse should be devised, with consideration to the limited capacity of most programmes to collect data. Providing mothers with MUAC tapes could facilitate collection of such data. This would be in line with the current field of thinking, as in 2017 The Council of Research and Technical Advice on Acute Malnutrition (CORTASAM) recommended that MUAC should be used as a primary admission criterion for SAM programmes [56]. Mobile phones within households or communities could be a useful tool for reporting relapse and mortality.

In order to ascertain a more complete evaluation of CMAM programmes, mortality and relapse 6 months post-discharge should be initiated as critical indicators of performance. In particular, post-treatment relapse rates and causal factors have been identified as a priority research area by the No Wasted Lives Coalition [57]. Conducting secondary analysis on existing high-quality programme data could aid the creation of a validated tool for use at discharge that systematically identifies children at highest risk of mortality and relapse. Routine referral to post-discharge nutrition-specific and nutrition-sensitive interventions such as the key Lancet evidence-based interventions, could be put in place for the most vulnerable children [58].

### Research recommendations

The dearth of literature in this area must be addressed, therefore conducting prospective cohort studies in a broader range of geographic and endemic disease settings in children post-SAM should be made a priority, alongside intervention studies exploring how to improve post-SAM outcomes and identify high-risk children. New studies should aim to provide a more comprehensive description of the problem, with particular focus on mortality and stunting. Mortality, stunting and relapse to SAM should be compared with sibling or community controls, alongside anthropometry, to give a more complete picture of the vulnerability of post-SAM children, and allow us to assess the attributable risk of these adverse outcomes to a previous episode of SAM. Furthermore, improved understanding of the risk factors that lead to relapse and excess mortality is needed. This can be achieved via case-control studies comparing relapsed with non-relapsed treated SAM children.

Randomised control trials are needed to determine interventions that can prevent relapse. One possibility is unconditional cash transfers, which were found to decrease the proportion of relapse to SAM following treatment in a recent study [38]. Determining whether this approach works in different settings and prevents relapse in the long-term should be made a priority. Another approach that needs urgent consideration is whether to treat SAM children with antibiotics after discharge from treatment. A recently published study suggests that treatment with antibiotics can reduce mortality in children even in the absence of malnutrition in different settings [59]. Children treated for SAM should be included in intervention studies that aim to improve HAZ through a variety of methods, including during adolescence [60]. Qualitative data should be collected to investigate and understand the role of the home environment and child feeding practices in children who relapse or die.

The increased risk of prevalence of chronic disease in children treated for SAM warrants further investigation. There is clearly a need to balance the short-term benefits of rapid weight gain with the possible long-term adverse consequences. Randomised control trials are required to explore the trade-off between slow and rapid weight gain. If the evidence suggests rapid weight gain is problematic, different treatment protocols should be tested with the aim of minimising future adverse consequences. A RUTF with a lower sugar content should be trialled in the light of the growing body of literature suggesting that high sugar foods can predispose to obesity [61].

As many studies in this review were excluded because they admitted on WAZ (See **S3 File**), admission criteria for future studies should be based on WHO definition of SAM, and discharge criteria on the WHO Protocol in order to standardise results. If studies choose to admit on WHZ, WAZ and HAZ, disaggregated data should be presented according to type of undernutrition, alongside age and sex.

As this review suggests, meeting anthropometric criteria for recovery may not equate to complete recovery and return to baseline risk of mortality and morbidity. The mortality and morbidity reported in studies suggests that children affected by an episode of SAM take longer to recover immunologically and physiologically than their weight gain would suggest and remain susceptible to problems like severe infection even after successful hospital discharge. Whether this is true for all ex-SAM children of a specific subset is important to determine in future work. Persisting vulnerability even after apparent nutritional cure may be explained by SAM being a symptom of other underlying problems (such as disability, malabsorption, immune dysfunction) rather than a random exposure, and calls for increased understanding of the aetiology. In order to increase our future understanding of SAM, future studies should report on a wider range of outcomes alongside mortality and relapse rates, such as body composition, cognition, behaviour, educational attainment, metabolism, immunity, and micronutrient deficiencies. This may enable us to broaden or re-define our definition of recovery in the future to one that, for example, could include immune and body composition parameters. This will allow us to determine when a child has truly returned to healthy physiological state, and also allows identification and further input to children who remain vulnerable to mortality and morbidity, despite being deemed recovered based on anthropometric criteria.

## Conclusions

Overall, there is a scarcity of studies that follow-up children 6–24 months post-discharge from SAM treatment. Limited data that exists suggests that children may exhibit sustained vulnerability even after achieving nutritional cure: this includes heightened mortality and morbidity risk and persistent stunting. Prospective cohort studies assessing a wider range of outcomes in children post-SAM are a priority as are intervention studies exploring how to improve post-SAM outcomes and identify high-risk children.

## Supporting information

S1 Checklist PRISMA checklist. (DOC)

S1 File Prospero registration details. (DOCX)

S2 File Search strategy. (DOCX)

S3 File Standardised Data extraction form. (DOCX)

S1 Table Table of studies excluded due to not meeting admission criteria. (DOCX)

S2 Table Quality assessment of studies. (DOCX)
