# Supplementary material for: Follow-up between 6 and 24 months after discharge from treatment for severe acute malnutrition in children aged 6-59 months: A systematic review
Source: PLoS One. 2018 Aug 30;13(8):e0202053. doi: 10.1371/journal.pone.0202053 (PMC6116928; doi:10.1371/journal.pone.0202053)
Supplement: S1 File — (DOCX) [file pone.0202053.s002.docx]

# PROSPERO Registration Details

**Publication number**: 42017065650

**Title**: Systematic review of the long-term follow-up of children aged 6-59 months discharged from treatment for severe acute malnutrition

**Date published**: 12/05/2017

**Available from**: http://www.crd.york.ac.uk/PROSPERO/display_record.asp?ID=CRD42017065650
