# Supplementary material for: Follow-up between 6 and 24 months after discharge from treatment for severe acute malnutrition in children aged 6-59 months: A systematic review
Source: PLoS One. 2018 Aug 30;13(8):e0202053. doi: 10.1371/journal.pone.0202053 (PMC6116928; doi:10.1371/journal.pone.0202053)
Supplement: S2 File — (DOCX) [file pone.0202053.s003.docx]

# Search Strategy and Results

MEDLINE In-Process & Other Non-Indexed Citations, searched 19/07/2107

Search # Term # Hits

001   "Democratic People's Republic of Korea"/ 167
002   (north korea or (democratic people* republic adj2 korea)).mp 398
003   Cambodia/ 2840
004   cambodia.mp.                                                    3932
005   Indonesia/                                                      8720
006   indonesia.mp 12448
007   Micronesia/                                                     1080
008   Kiribati.mp      141
009   Laos/                                                           1607
010   (laos or (lao adj1 democratic republic)).mp. 2336
011   marshall island*.mp.     281
012   Mongolia/                                                       1491
013   mongolia.mp. 3513
014   Myanmar/                                                       1771
015   (myanmar or burma).mp.                                          3230
016   Papua New Guinea/                                               3196
017   Papua New Guinea.mp. 4920
018   Philippines/                                                    7562
019   Philippines.mp. 10394
020   samoa/ or "independent state of samoa"/                          534
021   samoa.mp. 1000
022   Melanesia/                                                       976
023   Solomon Islands.mp. 678
024   Timor-Leste.mp.                                                  284
025   Tonga/                                                           235
026   tonga.mp.                                                        453
027   Vanuatu/                                                         315
028   Vanuatu.mp. 614
029   Vietnam/                                                       10524
030   Vietnam.mp.   15351
031   American Samoa/                                                  164
032   american samoa.mp. 349
033   exp China/                                                    141775
034   china.mp. 175919
035   Fiji/                                                          810
036   fiji.mp. 1506
037   Malaysia/                                                     13133
038   malaysia.mp. 17863
039   marshall islands.mp. 264
040   Palau/                                                           174
041   Palau.mp. 378
042   Thailand/                                                      23727
043   Thailand.mp. 31405
044   Tuvalu.mp. 50
045   Armenia/                                                        1291
046   Armenia.mp.   1630
047   Kosovo.mp. 776
048   Kyrgyzstan/                                             1222
049   (kyrgyzstan or kyrgyz republic or kirghizia or kirghiz).mp.     1498
050   Moldova/                                                         639
051   Moldova.mp. 880
052   Tajikistan/                                                      683
053   tajikistan.mp.                                                   878
054   Ukraine/                                                       15217
055   Ukraine.mp. 16519
056   Uzbekistan/                                                     1877
057   Uzbekistan.mp. 2158
058   Albania/                                                         742
059   Albania.mp. 1123
060   Azerbaijan/                                                     1140
061   Azerbaijan.mp.      1687
062   "Republic of Belarus"/                                          2010
063   (belarus or byelarus or belorussia).mp.     2535
064   Bosnia-Herzegovina/                                             1829
065   (bosnia or herzegovina).mp.     2637
066   Bulgaria/                                                       6076
067   Bulgaria.mp. 7410
068   "Georgia (Republic)"/                                         1558
069   Kazakhstan/                                                     2381
070   (Kazakhstan or kazakh).mp. 3270
071   "Macedonia (Republic)"/                                          462
072   Macedonia.mp.     1077
073   Montenegro/                                                      145
074   Montenegro.mp.       690
075   Romania/                                                        9342
076   Romania.mp. 11222
077   exp Russia/                                                    50708
078   USSR/                                                         42986
079   (Russia or Russian Federation or USSR or Union of Soviet Soc   91783
      ialist Republics or Soviet Union).mp.                      
080   Serbia/                                                         2452
081   serbia.mp. 4276
082   Turkey/                                                        30175
083   turkey.mp. not animal/     34899
084   Turkmenistan/                                                    561
085   Turkmenistan.mp.      713
086   Haiti/                                                          2819
087   Haiti.mp. 3610
088   86 or 87                                                        3610
089   Bolivia/                                                        2236
090   Bolivia.mp.     3461
091   El Salvador/                                                     807
092   El Salvador.mp. 1305
093   Guatemala/                                                      2691
094   Guatemala.mp.     3840
095   Honduras/                                                        996
096   Honduras.mp. 1661
097   Nicaragua/                                                      1321
098   Nicaragua.mp. 1990

099   Argentina/                                                    13385
100   Argentina.mp. 18766
101   Belize/                                                          520
102   Belize.mp. 830
103   Brazil/                                                        73578
104   Brazil.mp. 95337
105   Colombia/                                                       8102
106   Colombia.mp.    11706
107   Costa Rica/                                                     3263
108   Costa Rica.mp. 4841
109   Cuba/                                                           4752
110   Cuba.mp. 6255
111   Dominica/                                                         84
112   Dominica.mp. 401
113   Dominican Republic/                                             1366
114   Dominican Republic.mp.      2036
115   Ecuador/                                                        2955
116   Ecuador.mp. 4355
117   Grenada/                                                         116
118   Grenada.mp. 292
119   Guyana/                                                         610
120   Guyana.mp.                                                      1101
121   Jamaica/                                                        3335
122   Jamaica.mp. 4389
123   Mexico/                                                        33337
124   Mexico.mp. 51084
125   exp Panama/                                                     2182
126   Panama.mp. 4123
127   Paraguay/                                                        687
128   Paraguay.mp.                                                   1352
129   Peru/                                                          7055
130   Peru.mp. 10568
131   Saint Lucia/                                                     64
132   (St Lucia or Saint Lucia).mp. 322
133   "Saint Vincent and the Grenadines"/                               46
134   Grenadines.mp.        79
135   Suriname/                                                  827
136   Suriname.mp.     1033
137   Venezuela/                                                   4598
138   Venezuela.mp. 6479
139   Djibouti/                                                        203
140   (Djibouti or French Somaliland).mp. 358
141   Egypt/                                                         13807
142   Egypt.mp. 18097
143   Morocco/                                                        4913
144   Morocco.mp. 6578
145   Syria/                                                          1334
146   (Syria or Syrian Arab Republic).mp. 2200
147   Tunisia/                                                        7176
148   tunisia.mp.                                                     8786
149   Gaza.mp. 990
150   Yemen/                                                          1239
151   Yemen.mp. 1892
152   or/139-151                                                     37581
153   Algeria/                                                        2613
154   Algeria.mp. 3477
155   Iran/                                                          20623
156   Iran.mp. 34946
157   Iraq/                                                           4210
158   Iraq.mp. 8602
159   Jordan/                                                         3436
160   Jordan.mp. 5605
161   Lebanon/                                                     3539
162   Lebanon.mp. 4757
163   Libya/         1041
164   Libya.mp. 1512
165   Afghanistan/                                                    2807
166   Afghanistan.mp. 5642
167   Nepal/                                                          6495
168   Nepal.mp. 8851
169   Bangladesh/                                                     8868
170   Bangladesh.mp.                                                 12272
171   Bhutan/                                                          302
172   Bhutan.mp. 539
173   exp India/                                                91469
174   India.mp. 124364
175   Pakistan/                                                      14615
176   Pakistan.mp. 19378
177   Sri Lanka/                                                      5149
178   Sri Lanka.mp. 7012
179   Indian Ocean Islands/                                            700
180   Maldives.mp. 222
181   Benin/                                                          1302
182   (Benin or Dahomey).mp. 3405
183   Burkina Faso/                                                   2781
184   (Burkina Faso or Burkina Fasso or Upper Volta).mp. 3825
185   Burundi/                                                         590
186   Burundi.mp. 851
187   Central African Republic/                                        716
188   (Central African Republic or Ubangi-Shari).mp. 1067
189   Chad/                                                            621
190   Chad.mp. 1081
191   Comoros/                                                   256
192   (Comoros or Comoro Islands or Mayotte or Iles Comores).mp. 515
193   "Democratic Republic of the Congo"/                             3599
194   ((democratic republic adj2 congo) or belgian congo or zaire).mp. 3738
195   Eritrea/                                                        263
196   Eritrea.mp. 469
197   Ethiopia/                                                       9337
198   Ethiopia.mp. 12335
199   Gambia/                                                         2331
200   Gambia.mp. 3013
201   Guinea/                                                          855
202   (Guinea not (New Guinea or Guinea Pig* or Guinea Fowl)).mp.     3745
203   Guinea-Bissau/                                                   858
204   (Guinea-Bissau or Portuguese Guinea).mp. 1146
205   Liberia/                                                         999
206   Liberia.mp. 1591
207   Madagascar/                                                     2942
208   (Madagascar or Malagasy Republic).mp. 4495
209   Malawi/                                                         4206
210   (Malawi or Nyasaland).mp. 5987
211   Mali/                                                           2090
212   Mali.mp. 3392
213   Mozambique/                                                    1898
214   (Mozambique or Portuguese East Africa).mp. 3052
215   Niger/                                                          1047
216   (Niger not (Aspergillus or Peptococcus or Schizothorax or Cr    2944
      uciferae or Gobius or Lasius or Agelastes or Melanosuchus or
       radish or Parastromateus or Orius or Apergillus or Parastro
      mateus or Stomoxys)).mp.
217   Rwanda/                                                         1908
218   (Rwanda or Ruanda).mp. 2694
219   Senegal/                                                        5233
220   senegal.mp.                                                     6908
221   Sierra Leone/                                                  1225
222   Sierra Leone.mp.                                                1973
223   Somalia/                                                        1356
224   Somalia.mp.     1877
225   South Sudan/                                                     47
226   south sudan.mp.                                                  340
227   Tanzania/                                                       9642
228   (Tanzania or Zanzibar).mp. 12583
229   Togo/                                                        978
230   (Togo or Togolese Republic).mp. 1410     
231   Uganda/                                                        10073
232   Uganda.mp. 13393
233   Zimbabwe/                                                       5297
234   (Zimbabwe or Rhodesia).mp. 6829
235   Cameroon/                                                       4650
236   Cameroon.mp. 6540
237   Cape Verde/                                                      144
238   (Cape Verde or Cabo Verde).mp. 516
239   Congo/                                                          1630
240   (congo not ((democratic republic adj3 congo) or congo red).mp. 2316
241   Cote d'Ivoire/                                                 2826
242   (Cote d'Ivoire or Ivory Coast).mp. 3950
243   Ghana/                                                          6369
244   (Ghana or Gold Coast).mp. 9006
245   Kenya/                                                       13680
246   kenya.mp.                                                      18042
247   Lesotho/                                                         355
248   (Lesotho or Basutoland).mp. 603
249   Mauritania/                                                      391
250   Mauritania.mp. 612
251   Nigeria/                                                       25724
252   Nigeria.mp. 32071
253   Atlantic Islands/                                                721
254   (sao tome adj2 principe).mp. 115
255   Sudan/                                                          4410
256   (Sudan not south sudan).mp. [mp=title, abstract, original ti    7844
257   Swaziland/                                                       444
258   Swaziland.mp. [mp=title, abstract, original title, name of s     753
259   Zambia/                                                         3850
260   (Zambia or Northern Rhodesia).mp. 5213
261   Angola/                                                          823
262   Angola.mp. 1318
263   Botswana/                                                       1487
264   (Botswana or Bechuanaland or Kalahari).mp. 2266

265   Equatorial Guinea/                                               220
266   (Equatorial Guinea or Spanish Guinea).mp.                        400
267   Gabon/                                                          1339
268   (Gabon or Gabonese Republic).mp. 1899
269   Mauritius/                                                       505
270   (Mauritius or Agalega Islands).mp. 905
271   Namibia/                                                         867
272   Namibia.mp. 1396
273   South Africa/                                                  36861
274   South Africa.mp. 45484
275   ((developing or less* developed or under developed or 492

underdeveloped or middle income or low* income or

underserved or under served or deprived or poor*) adj

(economy or economies).ti,ab
276   ((developing or less* developed or under developed or 78983

underdeveloped or middle income or under served or

deprived or poor*) adj (countr* or nation? or population?

or world)).ti,ab
277   (low* adj (gdp or gnp or gross domestic or gross national)).ti,ab     203                                                  
278   (low adj3 middle adj3 countr*).ti,ab.                        8630
279   (lmic or lmics or third world or lami countr*).ti,ab.           5004
280   transitional countr*.ti,ab.                                      136
281   Developing Countries/                                          70600
282   "africa south of the sahara"/ or africa, central/ or africa, eastern/    18328

or africa, southern/ or africa, western/
283   ("africa south of the sahara" or sub-saharan africa or 31323
      central africa or eastern africa or southern africa or western
      africa).mp.                                                  
284   or/1-283                                                     1216790
285   (acute adj2 malnutrition).ti,ab.                                 908
286   (severe adj2 malnutrition).ti,ab.                               2347
287   "sever* acute* maln*".ti,ab.                                     444
288   "severe malnutrition".ti,ab.                                    1717
289   "acute malnutrition".ti,ab.                                      792
290   "acute severe malnutrition".ti,ab.                                13
291   "protein-energy malnutrition".ti,ab.                            2102
292   "protein-calori* malnutrition".ti,ab.                           1539
293   (undernutrition adj2 severe).ti,ab.                              207
294   (undernutrition adj2 acute).ti,ab.                                61
295   (severe* adj2 malnourish*).ti,ab.                               1239
296   (acute adj2 malnourish*).ti,ab.                                   16
297   (marasmic or marasmus).ti,ab.                                    934
298   kwas?io?kor.ti,ab.                                              1828
299   marasmic-kwas?io?kor.ti,ab.                                      179
300   (undernutrition or under-nutrition).ti,ab.                      6727
301   (undernourish* or under-nourish*).ti,ab.                        3331
302   (malnourish* or mal-nourish*).ti,ab.                            9497
303   (nutrition* adj1 deficien*).ti,ab.                              4470
304   (nutrition* adj1 disorder*).ti,ab.                              1253
305   (wasting or wasted).ti,ab.                                     17298
306   (mid* upper arm circumference adj2 "115").ti,ab.                  10
307   (mid* upper arm circumference adj2 "110").ti,ab.                   3
308   (MUAC adj2 "115").ti,ab.                                          25
309   (MUAC adj2 "110").ti,ab.                                           8
310   (nutrition* adj2 oedema*).ti,ab.                                  59
311   malnutrition/                                                  10909
312   nutritional disorder/                                          18541
313   nutritional deficiency/                                        10909
314   wasting syndrome/                                               1108
315   exp severe acute malnutrition/                                  2696
316   protein energy malnutrition/                                    7286
317   or/285-316                                                     68491
318   child*.ti,ab.                                                1200405
319   (infant or infancy).ti,ab.                                    185204
320   (preschool or pre-school or pre school).ti,ab.                 23961
321   toddler*.ti,ab.                                                 8377
322   infants/                                                      737497
323   children/                                                    1565516
324   318 or 319 or 320 or 321 or 322 or 323                       2338506
325   (follow up or follow-up or followup).ti,ab.                   816743
326   (long term or long-term or longterm).ti,ab.                   669508
327   (post-discharge or post discharge or postdischarge).ti,ab.      6580
328   surveillance.ti,ab.                                           139361
329   (after adj2 recover*).ti,ab.                                   43835
330   evaluat*.ti,ab.                                              2866832
331   longitudinal.ti,ab.                                           190999
332   longitudinal studies/                                       112006
333   aftercare/                                                      7625
334   or/325-333                                                   4149308
335   "ready to use therapeutic food".ti,ab.                           104
336   (therapeutic adj3 (food* or diet* or feed*)).ti,ab.             1890
337   (fortific* adj3 (food* or diet*)).ti,ab.                        1587
338   (enrich* adj3 (food* or diet*)).ti,ab.                          6174
339   (supplement* adj3 (food* or diet* or feed*)).ti,ab.            43599
340   (ready adj3 food*).ti,ab.                                        786
341   (RUTF or RTUT).ti,ab.                                            103
342   treat*.ti,ab.                                                4659031
343   intervention.ti,ab.                                           465489
344   (nutrition* adj2 (rehabilit* or recover*)).ti,ab.               1436
345   (manage* adj3 protocol).ti,ab.                                  3434
346   supplementary feeding/                                         14057
347   food, fortified/                                                8888
348   food, formulated/                                               6048
349   or/335-348                                                   5003187
350   284 and 317 and 324 and 334 and 349                             1100
351   limit 350 to humans 996
