# Supplementary material for: Follow-up between 6 and 24 months after discharge from treatment for severe acute malnutrition in children aged 6-59 months: A systematic review
Source: PLoS One. 2018 Aug 30;13(8):e0202053. doi: 10.1371/journal.pone.0202053 (PMC6116928; doi:10.1371/journal.pone.0202053)
Supplement: S3 File — (DOCX) [file pone.0202053.s004.docx]

# S3 File Data Extraction form

| **Title:**  **Author:**  **Year:**  **Journal:**  **Country:**  **Study Design:** |
| --- |
| **SAM** |
| **Age:** |
| **Number of participants:** |
| **Characteristics of participants (gender, ethnicity):** |
| **Intervention** |
| **Admission Criteria:** |
| **Inclusion/Exclusion criteria:** |
| **Setting (inpatient/outpatient/emergency):** |
| **Sampling/recruitment of participants:** |
| **Characteristics of setting (urban/rural):** |
| **Details of nutritional intervention:** |
| **Discharge criteria:** |
| **Follow-up** |
| **Follow-up details (length, no. visits etc.):** |
| **Number followed-up:** |
| **Results** |
| **Primary outcome:** |
| **Secondary outcomes:** |
| **Crude (& adjusted if available) effect size:** |
| **Methods of data collection:** |
| **Reliability/Validity/Blinding:** |
| **Control group used? Y/N**  **Details:** |
| **Confounding variables collected:** |
| **Strengths/Weaknesses:** |
| **Conflicts of interest? Y/N** |
| **Comments:** |
