# Supplementary material for: Follow-up between 6 and 24 months after discharge from treatment for severe acute malnutrition in children aged 6-59 months: A systematic review
Source: PLoS One. 2018 Aug 30;13(8):e0202053. doi: 10.1371/journal.pone.0202053 (PMC6116928; doi:10.1371/journal.pone.0202053)
Supplement: S1 Table — (DOCX) [file pone.0202053.s005.docx]

**File S3: Table of Studies Excluded due to not meeting admission criteria**

| Title, Author, Year, Study Design | N | Populations & Setting | Admission (A) & Discharge (D) Criteria | Description of intervention | Description of Follow-up | Outcomes reported & Findings |
| --- | --- | --- | --- | --- | --- | --- |
| Nutrition Rehabilitation in Hospital – a waste of time and money? Evaluation of Nutrition Rehabilitation in a rural district hospital in South-West Tanzania. II. Long-term results.  Van Roosmalen-Wiebenga, M. W et al.  1987 (1)  Retrospective cohort study | 475 | Children aged between 6 and ≥72 months admitted to the only hospital in southwestern Highlands of Tanzania between May 1980 and May 1983 | A1: ≤60% WAZ of National Center for Health Statistics (NCHS), **and/or:**  A2: Oedema  D: Oedema resolved, no infections, gaining weight and return of appetite and activity | Inpatient  Milk-based feeds  Nutrition education and counselling for caretakers | Between 6-36 months after discharge.  Outcomes presented as an average of these times. | **Loss-to-follow-up =** 141/475 (29.7%)  **Mortality =** 38/475 (8.0%)  **Relapse =** 62/475 (13.0%)  **Anthropometry** = Mean of NCHS reference median 1 year or more after leaving hospital   - Weight-for-age: 80.2 (9.4 SD) - Weight-for-height: 98.8 (8.2) - Height-for-age: 88.8 (4.1) |
| Long-term follow-up of severe protein-energy malnutrition in Eastern Zaire  Hennart,P et al.  1987 (2)  Prospective cohort study | 171 | Average at admission: 46 months | A1: Kwashiorkor (*serum albumin <3g/100ml)*  A2: Marasmus (*WHZ <5^th^ percentile of local growth curve)*  A3: Marasmic-kwashiorkor *(both signs)*  A4: Nutritional dwarfism (*height-for-age z-score (HAZ) <5^th^ percentile local growth curve)*  D: not reported | *Not reported* | Followed-up once a year for 5 years | **Mortality=**   - 26/171 (17.0%) at 1-year follow-up - 34/171 (19.9%) at 2-year follow-up |
| Treatment of persistent diarrhoea and malnutrition: Long-term effects of in-patient rehabilitation  Sullivan, PB.et al.  1991 (3)  Prospective Cohort Study | 22 | 6-36 months  Children recruited from the outpatient department of Medical Research Council’s Station in The Gambia | A1: weight-for-height <75% NCHS reference median  A2: Persistent diarrhoea ((≥4 loose stools per day for >2 weeks)  D: Cessation of diarrhoea for 5 days and steady weight gain | Inpatient care for 3-4 weeks with high-calorie fortified milk formula. Then transferred to outpatients. | At 1, 3, 6, and 12 months after admission.  Outcomes presented at 12 months follow-up | **Loss-to-follow-up=** 0/32 (0.0%)  **Mortality=** 2/32 (6.3%)  **Morbidity=**   - 7/22 (32.0%) had persistent/ intermittent diarrhoea   **Anthropometry=** Mean at discharge, 6 months, 12 months:   - Weight-for-height z-score (WHZ): -2.70, -2.40, -2.97 - Weight-for-age z-score (WAZ): -3.99, -3.77, -3.72 - MUAC (cm): 11.5, 11.9, 12.6 |
| A follow-up experience of 6 months of children with severe acute malnutrition in Dhaka, Bangladesh  Ashraf,H.et al.  2012 (4)  Prospective Cohort Study | 180 | 6-23 months  Attended day-care clinic in an urban area in Dhaka | A1: <-3 WHZ and/or <-3 WAZ with acute illness **and/or**  A2: Bipedal oedema  D: 80% weight-for-length of NCHS reference median | Treated at the clinic with milk-based diets from 08:00 to 17:00 every day.  When clinical improvement observed, transitioned to day-care nutrition rehabilitation unit | Follow-up assessment each week for 2 weeks, then every 2 weeks for 3 months, then monthly basis for 6 months.  Outcomes presented at 6 months | **Loss-to-follow-up**= 92/180 (51.1%)  **Mortality**= 5/180 (2.8%)  **Morbidity**=   - 9/180 (5.0%) diarrhoea - 28/180 (16%) cough - 24/180 (13.0%) fever   **Relapse**= 32/180 (17.8%)  **Anthropometry**= Mean at discharge (SD) & 6 months’ follow-up (SD)   - WAZ: -4.3 (0.9),   -3.4 (1.0)   - WHZ: -2.5 (0.6),   -1.9 (1.0)   - HAZ: -4.2 (1.3),   -3.9 (1.2) |
| Effects of psychosocial stimulation on growth and development of severely malnourished children in a nutrition unit in Bangladesh  Nahar,B.et al.  2009 (5)  Controlled intervention trial | 133 | 6-24 months | A1: Weight-for-age <50% NCHS reference median  A2: Weight-for-height <70% NCHS  A3: Bipedal-oedema  D: WHZ >80% NCHS & no oedema | Inpatient  All received nutritional care and an intervention group also received a psychosocial intervention (individual play sessions) | Follow-up visits at the hospital 7 times over 6 months post-discharge (twice in the first month then monthly) | **Loss-to-follow-up:**   - 6/43 (14.0%) in control group - 21/54 (38.9%) in intervention group   **Anthropometry**:  Both groups mean WAZ at discharge= -3.9 (1 SD)  At 6 months, control group WAZ increased to -3.6 (1.2 SD)  At 6 months intervention group WAZ increased to -3.1 (0.9 SD) |
| Abbreviations: HAZ= Height-for-age Z-score, MUAC = mid-upper arm circumference, NCHS = National Centre for Health Statistics, SD = Standard Deviation, WAZ = Weight-for-age Z-score, WHZ = weight-for-height Z-score | | | | | | |

1. Van Roosmalen-Wiebenga MW, Kusin JA, De With C. Nutrition rehabilitation in hospital: a waste of time and money? Evaluation of nutrition rehabilitation in a rural district hospital in south-west Tanzania. II. Long-term results. J Trop Pediatr. 1987;33(1):24-8.

2. Hennart P, Beghin D, Bossuyt M. Long-term follow-up of severe protein-energy malnutrition in eastern Zaire. J Trop Pediatr. 1987;33(1):10-2.

3. Sullivan PB, Mascie-Taylor CGN, Lunn PG, Northrop-Clewes CA, Neale G. The treatment of persistent diarrhoea and malnutrition: long-term effects of in-patient rehabilitation. Acta Paediatr. 1991;80(11):1025-30.

4. Ashraf H, Alam NH, Chisti MJ, Mahmud SR, Hossain MI, Ahmed T, et al. A follow-up experience of 6 months after treatment of children with severe acute malnutrition in Dhaka, Bangladesh. J Trop Pediatr. 2012;58(4):253-7.

5. Nahar B, Hamadani JD, Ahmed T, Tofail F, Rahman A, Huda SN, et al. Effects of psychosocial stimulation on growth and development of severely malnourished children in a nutrition unit in Bangladesh. Eur J Clin Nutr. 2009;63(6):725-31.

**References**
