# Supplementary material for: Follow-up between 6 and 24 months after discharge from treatment for severe acute malnutrition in children aged 6-59 months: A systematic review
Source: PLoS One. 2018 Aug 30;13(8):e0202053. doi: 10.1371/journal.pone.0202053 (PMC6116928; doi:10.1371/journal.pone.0202053)
Supplement: S2 Table — (DOCX) [file pone.0202053.s006.docx]

| Study | **External Validity Score (Section 1)** | **Internal Validity Score (Section 2-4)** |
| --- | --- | --- |
| Khare,RD.et-al^52^1976 | **+** | **-** |
| Khanum,S.et-al^54^1998 | **+** | **+** |
| Bahwere,P.et-al^51^2008 | **++** | **+** |
| Kerac,M.et-al^48^2014 | **-** | **++** |
| Aprameya,HS.et-al^49^2015 | **++** | **++** |
| Burza,S.et-al^50^2016 | **++** | **+** |
| Somasse,YE.et-al^53^2016 | **+** | **+** |
| (-) Poor-quality (+) Adequate-quality (++) Good-quality | | |

**S2 Table Quality Assessment of the Studies**
